# Efficiency of cervical cerclage and pessary in addition to vaginal progesterone to prevent preterm birth in twin pregnancies: a case-control study from a tertiary center

**DOI:** 10.55730/1300-0144.5968

**Published:** 2025-01-22

**Authors:** Göksun İPEK, Atakan TANAÇAN, Ilım DEMET, Zahid AĞAOĞLU, Ezgi BAŞARAN, Özgür KARA, Dilek ŞAHİN

**Affiliations:** 1 Division of Perinatology, Department of Obstetrics and Gynecology, Ankara Bilkent City Hospital, Ankara, Turkiye; 2 Department of Obstetrics and Gynecology, Ankara Bilkent City Hospital, Ankara, Turkiye

**Keywords:** Pessary, cerclage, twin pregnancy, preterm birth, cervical insufficiency

## Abstract

**Background/aim:**

This study evaluated the efficiency of cervical cerclage and pessary in addition to vaginal progesterone to prevent preterm birth in twin pregnancies.

**Materials and methods:**

This retrospective case-control study included 46 cases of twin pregnancy with cervical insufficiency delivered at Ankara Bilkent City Hospital between January 2022 and January 2024. Patients were grouped as those receiving cervical cerclage in addition to vaginal progesterone (n = 10), cervical pessary in addition to vaginal progesterone (n = 11), and only vaginal progesterone (n = 25). Patients’ data were obtained from the hospital’s database. Obstetric parameters (gravidity, parity, abortion, artificial reproductive technologies, second-trimester abortion) and ultrasound parameters (cervical length, intraamniotic sludge) were recorded. Gestational week at birth, latency period (diagnosis to delivery), and delivery after 34 weeks were evaluated for effectiveness. All parameters were compared between groups and evaluated for effectiveness as an independent factor for preterm birth.

**Results:**

The primary effectiveness parameters of latency period, birth week, and delivery after 34 weeks did not differ statistically between groups. When the parameters were evaluated independently of treatment groups for their effects on delivery after 34 weeks with multivariate regression analysis, the presence of intraamniotic sludge was found to be a negative independent factor for delivery after 34 weeks (p = 0.03).

**Conclusion:**

Cervical cerclage or pessary in addition to vaginal progesterone had no additional benefit for achieving birth after 34 gestational weeks. The only factor that had a negative effect on birth after 34 gestational weeks was the presence of intraamniotic sludge. Our clinical experience with twin pregnancies may provide insight into treatment options for clinicians.

## 1. Introduction

Cervical insufficiency (CI) is a condition characterized by the inability of the cervix to maintain a pregnancy in the second or early third trimester with painless cervical shortening, dilatation, and delivery. While the diagnosis is primarily made based on ultrasound results with cervical length of less than 25 mm measured by transvaginal ultrasound incidentally, history-based diagnoses can also be made in cases of recurrent second-trimester abortion/delivery [1,2]. The incidence of CI is approximately 1% among all pregnancies [3,4].

While multiple pregnancies alone are a determining risk factor for preterm birth, accompanying CI increases the risk of preterm delivery. Premature birth is one of the most important health problems in terms of neonatal mortality and morbidity [5].

Prophylactic and therapeutic treatment algorithms are more specific in singleton pregnancies and the treatment methods that may be effective in multiple pregnancies are not as clear. There are conflicting publications in the literature regarding the effectiveness of vaginal progesterone, cerclage, and pessary application, which are possible treatment methods in cases of singleton pregnancies.

In the literature, the use of cervical pessary was reported to be associated with the prolongation of twin pregnancies with cervical length of <15 mm [6]. Although there are some reports on the effectiveness of pessaries, cervical pessaries are generally not accepted as typical therapies in the literature and their therapeutic effects are unclear even for singleton pregnancies [5,7]. In a metaanalysis, ultrasound results indicated that cerclage was associated with an increase in the risk of preterm birth before 34 weeks of gestation in twin pregnancies [8]. On the contrary, in a few studies, cerclage before 24 weeks of gestation in twin pregnancies was found to be effective in reducing the risk of preterm birth [5,9]. There are difficulties in treatment selection and patient management in this high-risk patient group due to the conflicting results in the literature.

The aim of this study was to compare the effectiveness of different treatment modalities to prevent preterm delivery and to evaluate the factors that may affect premature birth in cases of twin pregnancies with CI.

## 2. Materials and methods

This case-control study was retrospectively conducted with the cases of women with dichorionic-diamniotic twin pregnancies diagnosed with CI who delivered in Ankara Bilkent City Hospital’s Department of Perinatology between January 2022 and January 2024. Patients who had cervical cerclage in addition to vaginal progesterone or used a cervical pessary in addition to vaginal progesterone to prevent preterm delivery constituted the case groups. All patients diagnosed with CI received vaginal progesterone treatment. Patients who refused any cervical interventions and used only vaginal progesterone constituted the control group used to compare cervical intervention efficiencies. The McDonald cerclage procedure was performed in all cerclage cases. All patients were informed about the procedures, therapy options, and treatment decisions. Patients’ data were obtained retrospectively from the hospital’s database and patient files.

The eligibility criteria for the study included having a dichorionic-diamniotic twin pregnancy, having no systemic chronic diseases, not using any medications, and having intact membranes at the time of diagnosis and cervical length data. Eligible patients were consecutively included in the study. For standardization of the groups, monochorionic-diamniotic twin pregnancies and other multiple pregnancies were excluded from the study. Patients with missing data and those with comorbidities, unclear diagnosis, or birth indications other than preterm labor were excluded from the study. Written informed consent was obtained from all participants. The study was approved by the Institutional Review Board of the University of Health Sciences Ankara Bilkent City Hospital Ethics Committee (Approval Number: E2-24-6139).

Maternal age and obstetric data (gravidity, parity, abortion and living children, history of artificial reproductive technologies, second-trimester abortion/delivery in previous pregnancies, and abortus imminens in present pregnancy) were recorded for all analyzed cases. Transvaginal sonographic cervical length at the time of diagnosis, diagnosis week, and indications (ultrasound-based/shortening, physical examination-based/dilatation, and history-based) were also evaluated. Gestational week at delivery, latency period (from diagnosis to delivery), and delivery after 34 weeks were analyzed to determine the effectiveness of cervical interventions in addition to vaginal progesterone use. Possible confounding parameters such as invasive procedures, tocolysis, early membrane rupture (24 h before delivery), and the presence of intraamniotic sludge at any time during the pregnancy were also noted for all analyzed cases. All parameters were compared between treatment groups and evaluated for their effectiveness as independent factors for preterm delivery. Antibiotic treatment was not given in cases of pregnancies with sludge in our clinic because there are conflicting results in the literature regarding the effectiveness of antibiotic treatment in such cases [10,11]. Patients who underwent cerclage were hospitalized for 3–5 days, and all patients were followed as outpatients weekly or every 2 weeks until delivery.

Statistical analyses were conducted using IBM SPSS Statistics 23 (IBM Corp., Armonk, NY, USA). Normality analyses were performed for the parameters of interest based on the Shapiro–Wilk test. Due to a lack of normal distribution, descriptive statistics were presented as median and minimum–maximum values. The Kruskal–Wallis test was used to compare parameters between groups. Categorical variables were given as numbers and percentages. The chi-square test was used to compare independent variables between groups. Multivariate regression analysis was used to investigate independent parameters. Statistical significance was defined as a two-tailed p-value of 0.05 with a 95% confidence interval.

## 3. Results

Ten patients were treated with cerclage in addition to progesterone, 11 were treated with a pessary in addition to progesterone, and 25 were treated with progesterone alone. Maternal age and obstetric parameters were similar between the groups. Use of artificial reproductive technology, second-trimester abortion/delivery history, cervical length, and week of diagnosis did not differ. Two of the cerclage cases entailed emergent rescue cerclage. One of 17 patients with abortus imminens history was in the cerclage group. All obstetric parameters are given in Table 1 with p-values.

Although the rate of delivery after 34 weeks was higher in the cerclage group than in the other groups, the primary effectiveness parameters of latency period, birth week, and delivery after 34 weeks did not differ statistically between the groups. Rates of the possible confounding factors of intraamniotic sludge and early membrane rupture were similar among the groups. These parameters are given with p-values in Table 2.

In the second part of the analysis, the parameters of interest were evaluated independently of treatment groups to determine their effects on delivery after 34 gestational weeks with multivariate regression analysis. The presence of intraamniotic sludge was found to be a negative independent factor for delivery after 34 weeks (p = 0.03). The results of multivariate regression analysis performed to investigate independent predictors of birth after 34 weeks are shown in Table 3.

## 4. Discussion

In this study, the effects of cervical pessary and cerclage applied in addition to vaginal progesterone on preterm birth in cases of dichorionic-diamniotic twin pregnancies with CI were investigated. It was found that neither cerclage nor pessary usage had any additional positive effect on birth after 34 weeks of gestation. Gestational weeks at birth and the latency period between week of diagnosis and birth were similar among the groups.

A recent metanalysis reported that vaginal progesterone in cases of twin gestation with short cervix length reduced the risk of preterm birth before 34 gestational weeks [12]. In a randomized controlled trial of twin pregnancies, the use of a cervical pessary was reported to be associated with a decrease in preterm birth in cases of short cervixes. On the contrary, in other studies, researchers did not demonstrate any benefits of using a cervical pessary in twin pregnancies [13,14], which is in line with the present results.

In previous metaanalyses, cerclage was found to be associated with a higher risk of preterm birth in twin pregnancies [8,15]. On the other hand, recent studies reported that cerclage in twin pregnancies was beneficial, especially in cases of cervical dilatation [16,17]. In the present study, cervical cerclage was ineffective in decreasing the preterm birth rate. A possible explanation for this result might be that patients in the present study were mostly ultrasound-indicated rather than dilatation-indicated, in contrast to previous studies.

In the present study, possible factors affecting preterm birth were examined independently of the treatment groups and the presence of intraamniotic sludge was found to be a risk factor for birth before 34 gestational weeks, similar to previous studies in the literature. In one previous study, the presence of intraamniotic sludge in twin pregnancies with short cervixes was found to be a risk factor for extreme prematurity [18]. In a different retrospective study, intraamniotic sludge was an independent risk factor for preterm birth in twin pregnancies [19].

The rates of history of artificial reproductive technology usage, abortus imminens, and second-trimester birth, evaluated as factors that may have affected patients’ treatment choices or gestational birth weeks, were similar among the groups analyzed in the present study. Transvaginal cervical lengths and gestational weeks at the time of diagnosis did not differ between groups. The diagnosis was most often made incidentally during a detailed ultrasound or at an outpatient clinic visit due to abortus imminens. In previous studies, short cervical length in combination with positive fetal fibronectin was reported to be associated with preterm birth in cases of twin pregnancies [19]. In the present study, fetal fibronectin was not evaluated together with cervical length measurements, and this difference in study design or the limited patient numbers may explain the discrepancies in results compared to previous studies in the literature.

This study was a comprehensive study examining the effectiveness of cervical treatment options in cases of dichorionic-diamniotic twin pregnancies with CI together with all other possible preterm birth risk factors. It is anticipated that this study may provide guidance for clinicians considering treatment options for twin pregnancies. Future studies with larger numbers of patients are needed to confirm the effectiveness of cervical interventions applied together with vaginal progesterone.

The possible limitations of this study include its retrospective design, limited patient numbers, and inability to evaluate fetal fibronectin. The strengths of the study were its comprehensive design, groups being carefully standardized compared to similar studies, and evaluation of the effects of parameters that could be confounding, which were evaluated independently of the treatment groups.

In conclusion, twin pregnancies diagnosed with CI constitute a high-risk group of patients, and preventing premature birth is of critical importance in terms of reducing neonatal morbidity and mortality. In this study, vaginal cerclage or pessary application in addition to vaginal progesterone did not have any negative effects other than increasing vaginal discharge. However, pessary and cerclage applications were found to have no additional benefits over progesterone treatment in achieving births after 34 weeks of gestation. The only factor that affected birth before 34 gestational weeks was the presence of intraamniotic sludge. Our clinical experience with twin pregnancies may provide insight into treatment options for clinicians in this field. More comprehensive studies are needed to guide clinical treatment selection in cases of twin pregnancies.

## Figures and Tables

**Table 1:** Comparison of obstetric parameters of the study groups.

Parameter	Progesterone and cerclage group (n = 10)	Progesterone and pessary group (n = 11)	Only progesterone group (n = 25)	p-value
Maternal age, (years)	29 (22–42)	31 (19–41)	30 (19–39)	0.97
Gravidity	1.5 (1–3)	1 (1–5)	2 (1–5)	0.53
Parity	0 (0–2)	0 (0–2)	0 (0–4)	0.27
Abortion	0 (0–2)	0 (0–2)	0 (0–2)	0.65
Living child	0 (0–2)	0 (0–2)	0 (0–4)	0.48
Abortus imminens, (%)	1 (10)	5 (45.5)	11 (44)	0.14
ART, (%)	4 (40)	4 (36.4)	9 (36)	0.97
2nd trimester loss, (%)	3 (30)	1 (9.1)	6 (24)	0.35
Cervical length, (mm)	15 (0–30)	14 (3–30)	16 (8–28)	0.74
Diagnosis time, (week)	22 (12–25)	22 (16–27)	22 (16–26)	0.49
Tocolysis, (%)	5 (50)	9 (81.8)	14 (56)	0.25
Invasive procedure (%)	4 (40)	1 (9.1)	8 (32)	0.47
Intramniotic sludge(%)	5 (50)	3 (27.3)	11 (44)	0.52

All parameters are given as median and minimum–maximum values due to the lack of normal distribution of the data. Categorical variables are shown as numbers and percentages. ART: Artificial reproductive technology.

**Table 2:** Comparison of study group outcomes.

Parameter	Progesterone and cerclage group (n = 10)	Progesterone and pessary group (n = 11)	Only progesterone group (n = 25)	p-value

Latency period, (week)	8.5 (4–23)	6 (2–15)	9 (1–15)	0.23

Delivery time, (week)	30.5 (21–37)	29 (22–36)	30 (22–36)	0.62

Indications, (%)				0.06
Ultrasound based	6 (60)	10 (90.9)	23 (92)	
Examination based	2 (20)	0 (0)	0 (0)	
History based	2 (20)	1 (9.1)	2 (8)	

EMR, (%)	1 (10)	4 (36.4)	7 (28)	0.36

> 34 weeks birth, (%)	4 (40)	2 (18.2)	5 (20)	0.40

All parameters are given as median and minimum–maximum values due to the lack of normal distribution of the data. Categorical variables are shown as numbers and percentages. EMR: Early membrane rupture.

**Table 3:** Results of multivariate regression analysis performed to investigate independent predictors of birth after 34 gestational weeks.

Variables	p-value	OR	95% confidence interval	
			Lower	Upper
Study groups	0.23	1.851	0.677	5.062
Cervical length	0.51	1.055	0.899	1.239
Diagnosis time, (week)	0.57	1.078	0.831	1.399
Intra-amniotic sludge	**0.03**	0.108	0.013	0.886
Invasive procedures	0.91	1.065	0.330	3.435
EMR	0.22	0.229	0.022	2.434
ART	0.19	0.223	0.023	2.166
Abortus imminence history	0.87	0.858	0.132	5.602

ART: Artificial reproductive technology; EMR: early membrane rupture.

## References

[1] Shennan A Story L Jacobsson B Grobman WA FIGO Working Group for Preterm Birth FIGO good practice recommendations on cervical cerclage for prevention of preterm birth International Journal of Gynecology and Obstetrics 2021 155 1 19 22 10.1002/ijgo.13835 PMC9291060 34520055

[2] Shennan AH Story L Royal College of Obstetricians, Gynaecologists Cervical Cerclage: Green-top Guideline No. 75 British Journal of Obstetrics and Gynaecology 2022 129 7 1178 1210 10.1111/1471-0528.17003 35199905

[3] Brown R Gagnon R Delisle MF No. 373-Cervical Insufficiency and Cervical Cerclage Journal of Obstetrics and Gynaecology Canada 2019 41 2 233 247 10.1016/j.jogc.2018.08.009 30638557

[4] Li J Jiang H Yao S Chen S Comparison of maternal and neonatal morbidity in transvaginal versus transabdominal cerclage patients: A retrospective study from two tertiary hospitals Taiwanese Journal of Obstetrics and Gynecology 2024 63 5 731 736 10.1016/j.tjog.2024.05.023 39266155

[5] Park JY Lee KN Kim HJ Choe K Cho A Pregnancy outcomes of cerclage in twin gestations: a multicenter retrospective cohort study The Journal of Maternal-Fetal and Neonatal Medicine 2024 37 1 2355495 10.1080/14767058.2024.2355495 38880661

[6] Jung DU Choi MJ Jung SY Kim SY Cervical pessary for preterm twin pregnancy in women with a short cervix Obstetrics and Gynecology Science 2020 63 3 231 238 10.5468/ogs.2020.63.3.231 32489967 PMC7231939

[7] ACOG Practice Bulletin No. 142: Cerclage for the management of cervical insufficiency Obstetrics and Gynecology 2014 123 2Pt1 372 379 10.1097/01.AOG.0000443276.68274.cc 24451674

[8] Saccone G Rust O Althuisius S Roman A Berghella V Cerclage for short cervix in twin pregnancies: systematic review and meta-analysis of randomized trials using individual patient-level data Acta Obstetricia et Gynecologica Scandinavica 2015 94 4 352 358 10.1111/aogs.12600 25644964

[9] Roman A Ramirez A Fox NS Prevention of preterm birth in twin pregnancies American Journal of Obstetrics and Gynecology MFM 2022 4 2S 100551 10.1016/j.ajogmf.2021.100551 34896357

[10] Cuff RD Carter E Taam R Bruner E Patwardhan S Effect of Antibiotic Treatment of Amniotic Fluid Sludge American Journal of Obstetrics and Gynecology MFM, 2020 2 1 100073 10.1016/j.ajogmf.2019.100073 33345987

[11] Hatanaka AR Franca MS Hamamoto TENK Rolo LC Mattar R Antibiotic treatment for patients with amniotic fluid “sludge” to prevent spontaneous preterm birth: A historically controlled observational study Acta Obstetricia et Gynecologica Scandinavica, 2019 98 9 1157 1163 10.1111/aogs.13603 30835813

[12] Romero R Conde-Agudelo A El-Refaie W Rode L Brizot ML Vaginal progesterone decreases preterm birth and neonatal morbidity and mortality in women with a twin gestation and a short cervix: an updated meta-analysis of individual patient data Ultrasound in Obstetrics and Gynecology 2017 49 3 303 314 10.1002/uog.17397 28067007 PMC5396280

[13] Nicolaides KH Syngelaki A Poon LC de Paco Matallana C Plasencia W Cervical pessary placement for prevention of preterm birth in unselected twin pregnancies: a randomized controlled trial American Journal of Obstetrics and Gynecology 2016 214 1 3e1 3.e39 10.1016/j.ajog.2015.08.051 26321037

[14] França MS Hatanaka AR Andrade VL Junior Elito Junior J Pares DBS Cervical Pessary Plus Progesterone for Twin Pregnancy with Short Cervix Compared to Unselected and Non-Treated Twin Pregnancy: A Historical Equivalence Cohort Study (EPM Twin Pessary Study) Revista Brasileira de Ginecologia e Obstetrícia 2020 42 10 621 629 10.1055/s-0040-1713806 33129217

[15] Berghella V Odibo AO To MS Rust OA Althuisius SM Cerclage for short cervix on ultrasonography: meta-analysis of trials using individual patient-level data Obstetrics and Gynecology 2005 106 1 181 189 10.1097/01.AOG.0000168435.17200.53 15994635

[16] Huang X Saravelos SH Li TC Huang R Xu R Cervical cerclage in twin pregnancy Best Practice and Research Clinical Obstetrics and Gynaecology 2019 59 89 97 10.1016/j.bpobgyn.2019.06.001 31331744

[17] Barbosa M Bek Helmig R Hvidman L Twin pregnancies treated with emergency or ultrasound-indicated cerclage to prevent preterm births The Journal of Maternal-Fetal and Neonatal Medicine 2020 33 19 3227 3232 10.1080/14767058.2019.1570119 30688138

[18] Boyer A Cameron L Munoz-Maldonado Y Bronsteen R Comstock CH Clinical significance of amniotic fluid sludge in twin pregnancies with a short cervical length American Journal of Obstetrics and Gynecology 2014 211 5 506e1 506.e5069 10.1016/j.ajog.2014.05.044 24881831

[19] Spiegelman J Booker W Gupta S Lam-Rochlin J Rebarber A The Independent Association of a Short Cervix, Positive Fetal Fibronectin, Amniotic Fluid Sludge, and Cervical Funneling with Spontaneous Preterm Birth in Twin Pregnancies American Journal of Perinatology 2016 33 12 1159 1164 10.1055/s-0036-1585582 27434692

